# Sexual Health Influencer Distribution of HIV/Syphilis Self-Tests Among Men Who Have Sex With Men in China: Secondary Analysis to Inform Community-Based Interventions

**DOI:** 10.2196/24303

**Published:** 2021-06-01

**Authors:** Nancy Yang, Dan Wu, Yi Zhou, Shanzi Huang, Xi He, Joseph Tucker, Xiaofeng Li, Kumi M Smith, Xiaohui Jiang, Yehua Wang, Wenting Huang, Hongyun Fu, Huanyu Bao, Hongbo Jiang, Wencan Dai, Weiming Tang

**Affiliations:** 1 University of Hawai'i John A. Burns School of Medicine Hawai'i, HI United States; 2 Dermatology Hospital of Southern Medical University Guangzhou China; 3 University of North Carolina Project-China Guangzhou China; 4 Institute of Global Health and STI Research of Southern Medical University Guangzhou China; 5 Department of Clinical Research, London School of Hygiene & Tropical Medicine London United Kingdom; 6 Zhuhai Center for Diseases Control and Prevention Zhuhai China; 7 Zhuhai Xutong Voluntary Services Center Zhuhai China; 8 Division of Epidemiology and Community Health, The University of Minnesota Twin cities Minneapolis, MN United States; 9 Division of Community Health and Research, Eastern Virginia Medical School Norfolk, VA United States; 10 Department of Epidemiology and Biostatistics, School of Public Health, Guangdong Pharmaceutical University Guangzhou China

**Keywords:** sexual health influencer, men who have sex with men, HIV, syphilis, self-test, sexual health, influencer, social network, peers

## Abstract

**Background:**

Social network–based strategies can expand HIV/syphilis self-tests among men who have sex with men (MSM). Sexual health influencers are individuals who are particularly capable of spreading information about HIV and other sexually transmitted infections (STIs) within their social networks. However, it remains unknown whether a sexual health influencer can encourage their peers to self-test for HIV/syphilis.

**Objective:**

The aims of this study were to examine the impact of MSM sexual health influencers on improving HIV/syphilis self-test uptake within their social networks compared to that of nonsexual health influencers.

**Methods:**

In Zhuhai, China, men 16 years or older, born biologically male, who reported ever having had sex with a man, and applying for HIV/syphilis self-tests were enrolled online as indexes and encouraged to distribute self-tests to individuals (alters) in their social network. Indexes scoring >3 on a sexual health influencer scale were considered to be sexual health influencers (Cronbach α=.87). The primary outcome was the mean number of alters encouraged to test per index for sexual health influencers compared with the number encouraged by noninfluencers.

**Results:**

Participants included 371 indexes and 278 alters. Among indexes, 77 (20.8%) were sexual health influencers and 294 (79.2%) were noninfluencers. On average, each sexual health influencer successfully encouraged 1.66 alters to self-test compared to 0.51 alters encouraged by each noninfluencer (adjusted rate ratio 2.07, 95% CI 1.59-2.69). More sexual health influencers disclosed their sexual orientation (80.5% vs 67.3%, *P*=.02) and were community-based organization volunteers (18.2% vs 2.7%, *P*<.001) than noninfluencers. More alters of sexual health influencers came from a rural area (45.5% vs 23.8%, *P*<.001), had below-college education (57.7% vs 37.1%, *P*<.001), and had multiple casual male sexual partners in the past 6 months (25.2% vs 11.9%, *P*<.001).

**Conclusions:**

Being a sexual health influencer was associated with encouraging more alters with less testing access to self-test for HIV/syphilis. Sexual health influencers can be engaged as seeds to expand HIV/syphilis testing coverage.

## Introduction

A social network is a network of individuals connected by interpersonal relationships [1]. Social network–based interventions are promising for promoting HIV testing and sexual health [2,3], including among men who have sex with men (MSM) who are at higher risk of HIV and other sexually transmitted infections (STIs) [4-10]. Social network–based strategies can increase testing access [11-14] and mitigate the stigma preventing MSM from seeking health care [10,15]. Many studies have found that individuals at the center of their networks can encourage health behavior change among their peers [16]. However, there is limited research on whether social influencers are better than noninfluencers at promoting HIV testing. A previous study found that MSM with more sexual health influence had greater engagement in sexual health campaigns and adoption of HIV/syphilis testing [17], but did not examine the interaction between influential MSM and their social network.

Strategies are needed to identify influential individuals for effective network dissemination of HIV/STI interventions. We define “sexual health influencers” as individuals whose sexual health knowledge and behaviors are more likely to influence, than be influenced by, peers in their social network, based on a prior MSM sexual influence study in China [17]. Although the precise criteria for “peer” differs among individual studies, peers are generally understood to be individuals that share key characteristics such as sexual orientation [18]. In this context, we consider sexual health influencers to be a subset of peers that are selected as peer educators based on their preexisting influence and social ties. Sexual health influencers are different from popular opinion leaders (POLs), a commonly used model in peer education interventions. POLs are rigorously trained for influencing their target audience, with whom they may not have prior social ties even if they are peers [2]. There is evidence that the effectiveness of POLs [4-8] is predicated on preexisting influence, and POL interventions that do not consider preexisting influence are less likely to succeed [19]. Additionally, interventions that do not engage influential individuals for dissemination may exclude hard-to-reach individuals [3] such as those with limited health care knowledge and access, and those who do not identify with at-risk communities but practice at-risk behaviors.

Our previous implementation program used a secondary distribution strategy to promote HIV/syphilis self-testing among Chinese MSM [20]. Secondary distribution is a social network–based strategy that involves giving one individual (index) multiple self-testing kits for distribution to their social contacts (alters) [12]. Evaluation of this implementation program demonstrated that secondary distribution can expand HIV/syphilis self-testing among MSM in a middle-income country [20]. Nonetheless, it remains unexplored whether sexual health influencer status is associated with greater promotion of HIV/STI self-testing among peers, especially peers who have less access to testing.

The primary objective of this study was to evaluate whether sexual health influencer MSM are more effective at encouraging HIV/STI self-testing among their peers compared to noninfluencers. Additionally, we aimed to test the hypothesis that sexual health influencers can reach alters in greater need of HIV/STI testing than noninfluencers. Our findings can improve network-targeting strategies for distributing HIV and syphilis self-tests, and increase access to sexual health interventions for MSM.

## Methods

### Participants and Enrollment

This was a retrospective cohort analysis of data obtained from an implementation study performed in Zhuhai, China. Detailed methods were described previously [20]. We partnered with Zhuhai Xutong MSM Service Center (hereafter “Xutong”), a gay community-based organization (CBO) based in Zhuhai, China, to use their HIV/syphilis self-test (hereafter “self-test”) distribution platform on WeChat (China’s largest social media platform). Men who applied for self-tests on this platform were invited to participate in our study. Participants were enrolled as indexes if they were 16 years or older, born biologically male, ever had sex with a man, applied for at least one self-test during the study period, and willing to complete a follow-up survey. Following provision of informed consent online, indexes completed a baseline survey, and then provided up to five self-tests per application for a deposit of US $14.70 per self-test. Multiple applications were allowed. Indexes were encouraged to use the self-tests not only for themselves but to also distribute self-tests to individuals in their social networks (hereafter “alters”). Each self-test contained a unique QR code for anonymous upload of a test result photograph and a follow-up survey. Upon upload of results, the tester was given a US $3.00 incentive, and the deposit associated with that self-test was refunded to the index through WeChat. Additionally, alters were asked to report their self-test experience. Alters of all sexes, genders, and sexual orientation were included.

Prior to study implementation, the surveys were pilot-tested with a small group of representatives from our partner MSM community. Surveys and participant responses were stored on the secure survey platform Wenjuanxing (Sojump, Shanghai, China) protected with passcodes accessible only to the research team.

### Identifying Sexual Health Influencers

Indexes were categorized as sexual health influencers or noninfluencers based on their responses to a 6-item sexual health influencer scale in the baseline survey. Each item was scored on a 5-point Likert-type scale, with a higher score indicating that the index is more likely to influence their peers and a lower score indicating that the index is more likely to be influenced by peers (Multimedia Appendix 1). Items were adapted from a scale previously studied in Chinese-speaking MSM populations [6,17]. Indexes were categorized as sexual health influencers if their mean score was greater than 3, using the same cut-off that previously identified sexual health influencers in a nationwide sample of MSM in China [17]. Cronbach α was .87 for the sexual health influencer scale in this study.

### Survey Measures

We asked participants about their sociodemographic characteristics, including age, residence registration (rural or urban), sexual orientation, educational attainment, and monthly income. We also asked participants about their health behaviors, including disclosure of sexual orientation, number of male partners in the past 6 months, and prior HIV testing. For indexes, we identified those who were MSM CBO volunteers based on a list of volunteers from Xutong. For alters, we also asked whether they tested simultaneously with the index.

### HIV and Syphilis Self-Test Results

All participants were asked to report their self-test results and upload a result photograph for verification. All results were reviewed by trained Xutong volunteers who followed up as needed for results verification or linkage to care. Only newly positive cases were counted in this study.

### Linkage of Indexes and Alters

**Figure 1:**
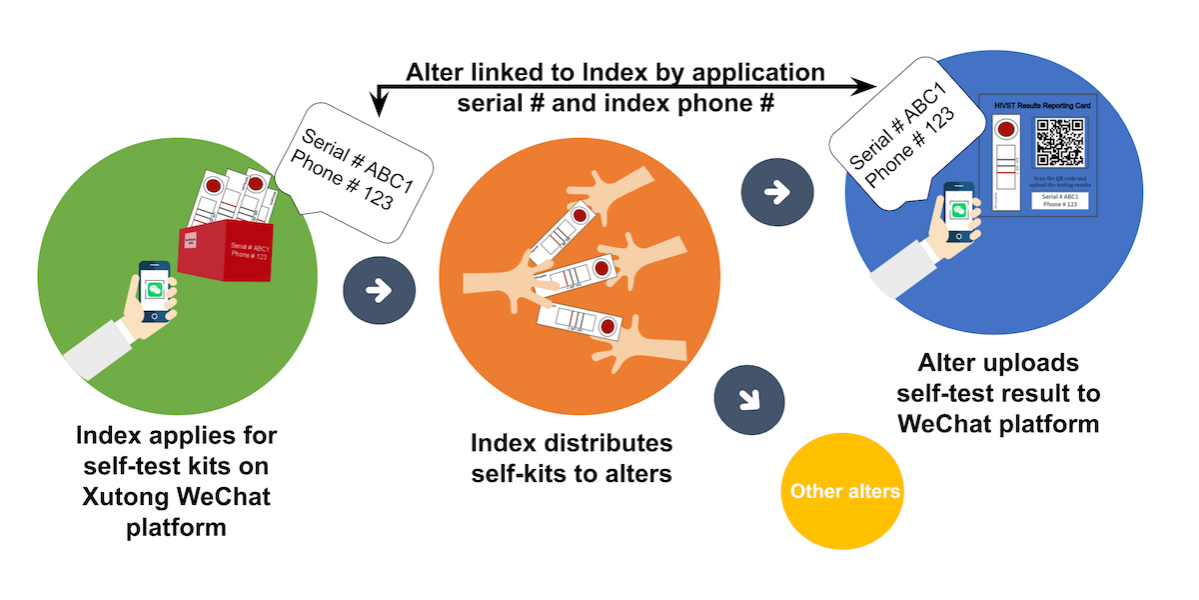
HIV/syphilis self-tests application, distribution, and linkage of alters to indexes.

Each applying index had to report their phone number and was assigned a unique application code. Participants uploading a self-test result were asked to report the test kit application phone number and code, which were used to link alters and indexes (Figure 1). Alters not linked to an enrolled index were excluded from analysis.

### Outcomes

The primary outcome was whether sexual health influencers could encourage more alters to self-test compared to noninfluencers. Between sexual health influencers and noninfluencers, we compared the mean number of applications submitted, self-tests obtained, and alters who reported a self-test result. We controlled for the following potential confounders: income, disclosed sexual orientation, CBO volunteer status, and prior HIV testing.

Secondary outcomes included the following: among sexual health influencers versus noninfluencers who distributed to at least one alter, we compared the mean number of alters who were first-time testers, performed simultaneous tests with the index, and alters with HIV-reactive or syphilis-reactive tests. Volunteer status of the index was selected a priori as a confounder. We also compared the characteristics of alters who received a self-test from sexual health influencers versus noninfluencers.

### Statistical Analysis

Descriptive analyses were performed for the sociodemographic and behavioral characteristics of the index and alter participants. The characteristics of sexual health influencers and noninfluencers, as well as characteristics of their respective alters, were compared using *t* tests and χ^2^ tests. Poisson regression was used to estimate the ratio of distribution by sexual health influencers versus noninfluencers, which are reported as the adjusted rate ratio (aRR) and 95% CI. Additional variables were added to the regression to control for confounders. Statistical analyses were performed using SAS Version 9.4.

### Ethical Statement

Prior to launching the study, ethical approval was obtained from the institutional review board at Zhuhai Municipal Center for Diseases Prevention and Control in China (ZHCDC2018014).

## Results

### Data Collection

**Figure 2:**
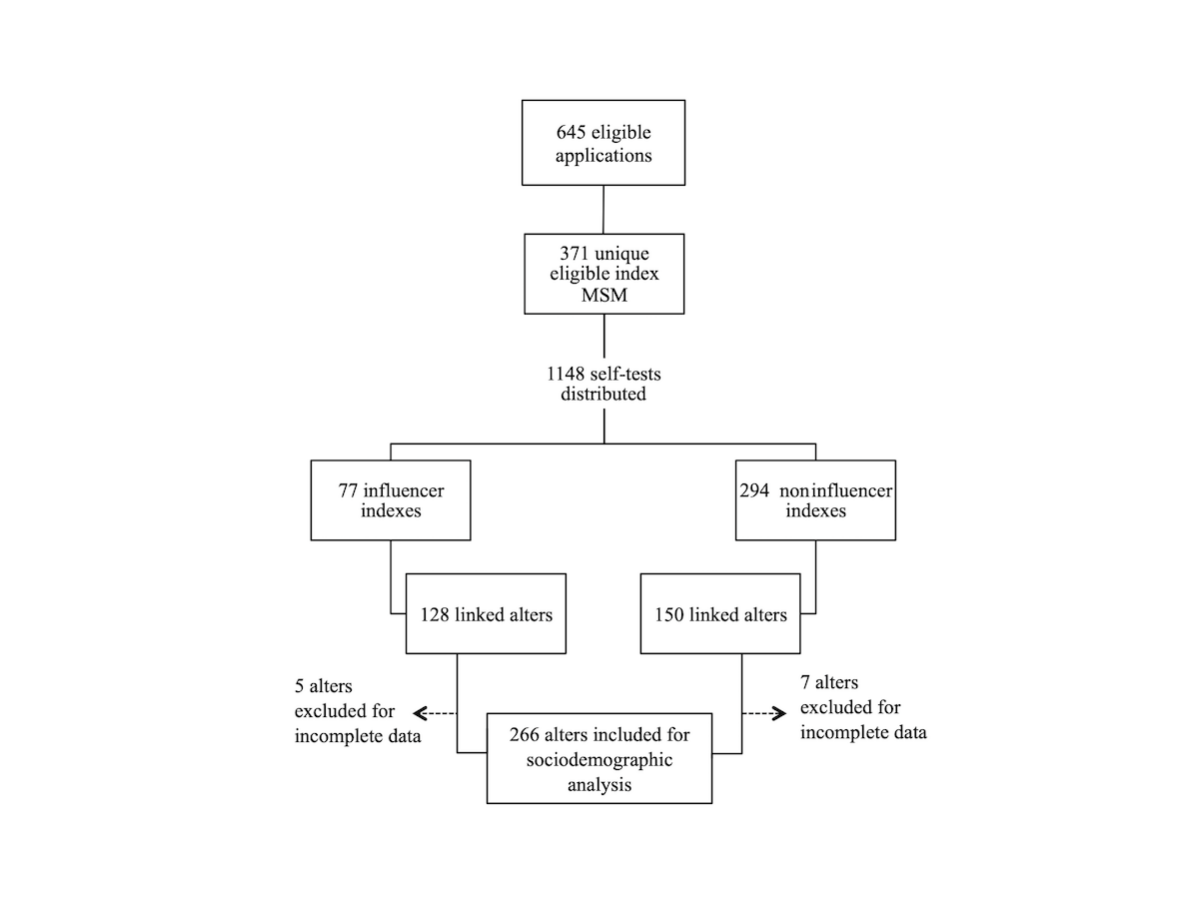
Flowchart of study participants.

Data were collected between June 17, 2018 and November 12, 2019. During this period, 371 unique indexes applied for 1148 self-tests, for which 1099 self-test results were returned by indexes and 278 unique alters linked to enrolled indexes. Of the alters, 266 completed the sociodemographic portion of the survey (Figure 2).

### Characteristics of Index Participants

Of the 371 indexes, 77 (20.8%) were sexual health influencers and 294 (79.2%) were noninfluencers. The distribution of sexual health influencer scores is provided in Multimedia Appendix 2. Characteristics of sexual health influencers and noninfluencers were largely similar but with some key differences. More sexual health influencers had disclosed their sexual orientation (80.5% vs 67.3%, *P*=.02) and were MSM CBO volunteers (18.2% vs 2.7%, *P*<.001) compared to noninfluencers. Additionally, more sexual health influencers had prior HIV testing compared to noninfluencers, although the difference was not statistically significant. Index participant characteristics are summarized in Table 1.

**Table 1:** Sociodemographic and health behavioral characteristics of the index participants in China, 2018-2019.

Characteristic			All indexes (N=371)		Sexual health influencers (n=77)		Noninfluencers (n=294)		*P* value
Age (years), mean (SD)			29 (6.9)		30 (8.2)		28 (6.5)		.06
**Residence, n (%)**									.79
	Rural	164 (44.2)		33 (42.9)		131 (44.6)			
	Urban	207 (55.8)		44 (57.1)		163 (55.4)			
**Education, n (%)**									.82
	Less than college	173 (46.6)		35 (45.5)		138 (46.9)			
	College or more	198 (53.4)		42 (54.6)		156 (53.1)			
**Annual income (US $)^a^, n (%)**									.41
	<8393	189 (50.9)		36 (46.8)		153 (52.0)			
	≥8393	182 (49.1)		41 (53.3)		141 (48.0)			
**Sexual orientation, n (%)**									.62
	Gay	259 (69.8)		52 (67.5)		207 (70.4)			
	Bisexual/other	112 (30.2)		25 (32.5)		87 (29.6)			
**Disclosed sexual orientation, n (%)**									.02
	Disclosed	260 (70.1)		62 (80.5)		198 (67.3)			
	Undisclosed	111 (29.9)		15 (19.5)		96 (32.7)			
**CBO^b^ volunteer, n (%)**									<.001
	Yes	22 (5.9)		14 (18.2)		8 (2.7)			
	No	394 (94.1)		63 (81.8)		286 (97.3)			
**Ever tested for HIV, n (%)**									.21
	Yes	294 (79.3)		65 (84.4)		229 (77.9)			
	No	77 (20.8)		12 (15.6)		65 (22.1)			
**>1 casual male partner in past 6 months, n (%)**									.31
	Yes	117 (31.5)		28 (36.4)		89 (30.3)			
	No	254 (68.5)		49 (63.6)		205 (69.7)			

^a^In 2017, the average annual income in China was US $7712 (National Bureau of Statistics China).

^b^CBO: community-based organization.

### Rates of Index Self-Test Distribution and Results Return

On average, each sexual health influencer index had twice as many alters who returned a test result compared to each noninfluencer (aRR 2.07, 95% CI 1.59-2.69). Each sexual health influencer also submitted more applications (aRR 1.32, 95% CI 1.10-1.58) and requested more self-tests (aRR 1.40, 95% CI 1.22-1.60) than each noninfluencer.

When the analysis was limited to indexes with at least one alter who returned a test, sexual health influencers distributed to more alters without prior HIV testing compared to noninfluencers, but this difference was not significant after controlling for the volunteer status of the index (aRR 1.28, 95 CI% 0.85-1.92). Sexual health influencers also performed more simultaneous testing with their alters, and distributed to more alters with HIV-reactive and syphilis-reactive test results compared to noninfluencers, but these differences were not statistically significant (Multimedia Appendix 3 and Multimedia Appendix 4).

During analysis, one outlier sexual health influencer was found to have distributed to 37 alters. To account for potential skew, data from this outlier were excluded in repeat analysis. Differences between sexual health influencers and noninfluencers in the self-test distribution remained the same after excluding this “superdistributer” outlier (see Multimedia Appendix 5 and Multimedia Appendix 6).

### Comparing the Alters of Sexual Health Influencers and Noninfluencers

Alters of sexual health influencers versus noninfluencers had some key differences. More alters of sexual health influencers were registered with a rural residence (45.5% vs 23.8%, *P<*.001) and had below college-level education (57.7% vs 37.1%, *P*<.001) compared to alters of noninfluencers. Additionally, more alters of sexual health influencers had more than one casual male partner in the past 6 months (25.2% vs 11.9%, *P*=.005) compared to alters of noninfluencers. Detailed characteristics of the alter participants are provided in Table 2.

**Table 2:** Characteristics of responding alter participants in Zhuhai, China, 2018-2019.

Characteristic		All alters (N=266)	Alters of sexual health influencers (n=123)	Alters of noninfluencers (n=143)	*P* value
Age (years), mean, SD		29 (7.0)	28 (6.6)	30 (7.3)	.08
**Sex at birth, n (%)**					.13
	Male	262 (98.5)	123 (100.0)	139 (97.2)	
	Female	4 (1.5)	0 (0.0)	4 (2.8)	
**Residence, n (%)**					<.001
	Rural	90 (33.8)	56 (45.5)	34 (23.8)	
	Urban	176 (66.2)	67 (54.5)	109 (76.2)	
**Education, n (%)**					<.001
	Less than college	124 (46.6)	71 (57.7)	53 (37.1)	
	College or more	142 (53.4)	52 (42.3)	90 (62.9)	
**Sexual orientation, n (%)**					.87
	Gay	176 (68.2)	82 (66.7)	94 (65.7)	
	Bisexual/other	90 (33.8)	41 (33.3)	49 (34.3)	
**>1 male casual partner in past 6 months, n (%)**					.005
	Yes	48 (18.1)	31 (25.2)	17 (11.9)	
	No	218 (82.0)	92 (74.8)	126 (88.1)	

## Discussion

### Principal Findings

Our implementation study demonstrated that in China, being an MSM sexual health influencer was associated with encouraging more alters to self-test for HIV/syphilis than being a noninfluencer. More importantly, sexual health influencers were associated with alters from rural regions of China and with less education, factors associated with limited access to HIV testing [21,22]. Alters of sexual health influencers also sought more casual sexual partners and thus were at greater risk of HIV infection [23]. These findings suggest that sexual health influencers and noninfluencers can reach different groups of individuals for testing. Our study extends the existing literature on HIV/syphilis self-test distribution by examining the role of sexual health influence. Our findings can inform future studies to increase the reach of self-tests for MSM and other populations at risk of HIV.

We found that being a sexual health influencer was associated with encouraging more alters to self-test than being a noninfluencer. This is consistent with prior findings that MSM peer leaders selected for their social influence were more effective at increasing HIV testing than nonpeer-led interventions [6-8,15,24]. Moreover, sexual health influencers applied for more self-tests than noninfluencers, suggesting greater engagement in self-test promotion. Although being a sexual health influencer was associated with encouraging more first-time testers to test, this was confounded by CBO volunteer status, suggesting that being a volunteer also affected self-test distribution capacity. CBO volunteers have experience in peer engagement even if they do not have other socially influential traits. Our results indicate that the self-administered sexual health influencer scale [17] can identify influential MSM to expand the reach of HIV and syphilis self-tests, and training can help them reach subgroups at higher risk such as MSM without prior testing.

Being a sexual health influencer was also associated with reaching more alters with lower education, from a rural residence, and with more casual sexual partners. These findings suggest that sexual health influencers could better reach individuals at greater risk of HIV but with less access to health care. Several studies have found alters to reach more MSM at high risk of HIV/STIs with low access to testing compared to other recruitment strategies [9,25,26]. Highly influential MSM may help reach individuals with the least access to HIV care and who are often missed by random seeding of interventions [3]. Our findings indicate that self-identified sexual health influencers are low cost and may be effective seeds for HIV/syphilis self-test distribution.

In this study, sexual health influencers were more likely to have disclosed their sexual orientation and to be CBO volunteers compared to noninfluencers. This is consistent with prior findings that MSM sexual health influencers were more likely to disclose their sexual orientation and have greater community engagement [17]. Shared experience between influencers and their target audience (ie, sexual orientation) is known to contribute to the effectiveness of interventions [18]. Moreover, volunteer experience and disclosed sexual orientation may contribute to greater social influence. These findings reinforce the correlation between sexual health influence and volunteerism among MSM, which may contribute to self-test distribution capability. The sexual health influencer scale identified many MSM who were not volunteers but nonetheless had influential qualities and were associated with greater self-test distribution capability, suggesting that sexual health influencers can expand an existing pool of volunteers and reach into untapped social networks.

Although we did not collect data on why sexual health influencers are effective at promoting HIV/syphilis testing, we propose some mechanisms based on existing theories underlying the mechanisms of peer-based health interventions. Our sexual health influencer scale selected for MSM who are socially visible and are sought by peers for advice and information. Based on social comparison theory, sexual health influencers may act as models of health and self-improvement in the MSM community, providing an upward comparison to which other MSM aspire [18]. Sexual health influencers are also experienced in providing social support, which suggests that they may be skilled at providing calm and reducing stress [18] while promoting health behaviors that can cause significant distress for the alter MSM. Few studies on network-based sexual health interventions describe theoretical mechanisms for their efficacy [3]. Future studies should elicit mechanisms that demonstrate the effectiveness of sexual health influencers.

### Limitations

Our study has several limitations. First, this was a secondary analysis, which identified sexual health influencers retrospectively. We found correlations between sexual health influence and self-test distribution, but other unaccounted variables may explain the different rates of distribution, such as specific characteristics of an index’s social network, influential characteristics unrelated to sexual health, and geographic access between the index and alters. Analyses for alters and indexes were also performed retrospectively, and we could not capture alters who received a self-test but did not return their results. This may have resulted in an underestimation of index self-test distribution rates. Nonetheless, the higher rate of test result through sexual health influencer return may indicate better linkage to care compared to that through noninfluencers. Second, participants were recruited online and required access to the internet for follow-up. Thus, our study only captured MSM with internet access and may have limited representativeness of MSM who do not use online social media. Third, our study was implemented through a well-established gay CBO in a populous city using their existing and popular self-testing platform. This lent our study credibility from participants but limits the generalizability of our findings. Our study may exclude MSM who are not part of any MSM network, and may not be applicable to some regions such as rural communities with weaker MSM networks. Finally, the sexual health influencer scale has only been studied among MSM in East Asia to date, and may have limited generalizability to other cultural and linguistic settings.

### Implications

Our study has implications for future HIV/syphilis self-test research and implementation. Primarily, we identified influential individuals using a simple, self-report sexual health influencer scale. This is important when considering the resource intensiveness and complexity of most social network interventions [16]. Simplicity allows for greater sustainability, especially where resource constraints limit social network mapping. Self-identification is an established, low-cost strategy for defining preexisting social influence, but is infrequently utilized due to concerns of subjectivity [27,28]. Nonetheless, we found self-reported sexual health influence to correlate with objective advantages. The sexual health influencer scale can be easily adopted by community programs to identify effective health promoters and educators, particularly when introducing a new health behavior to a marginalized community such as MSM. Our findings indicate social influence to be a core component of effective sexual health promotional campaigns, which should be adopted in sexual health promotion policies. Future randomized controlled studies should test the sexual health influencer scale as an intervention to promote HIV self-testing.

### Conclusions

Our study indicates that sexual health influencers are important for encouraging social network–based HIV/syphilis testing. Our findings are notable for the greater distribution by sexual health influencers compared to noninfluencers and increasing access to MSM linked to rural regions, where gay venues and health care facilities may be less accessible, and to MSM with lower education and at higher risk of HIV. Our study can inform future implementation research on social network targeting for HIV self-testing and sexual health interventions.

## Appendix

Multimedia Appendix 1 The six-item sexual health influencer scale.

Multimedia Appendix 2 Distribution of sexual health influence score and distribution statistics for index participants.

Multimedia Appendix 3 Self-test application and distribution by sexual health influencer and noninfluencer index men who have sex with men in Zhuhai, China, 2018-2019 (N=371).

Multimedia Appendix 4 Self-testing behaviors and outcomes of alters reached by sexual health influencer and noninfluencer index men who have sex with men in Zhuhai, China, 2018-2019 (N=116). Data include only indexes with at least one alter who returned a test result.

Multimedia Appendix 5 Self-test application and distribution by sexual health influencer and noninfluencer index men who have sex with men in Zhuhai, China, 2018-2019 excluding 1 superdistributor (outlier) influencer (N=370).

Multimedia Appendix 6 Self-testing behaviors and outcomes of alters reached by sexual health influencer and noninfluencer index men who have sex with men in Zhuhai, China, 2018-2019 excluding 1 superdistributor (outlier) influencer (N=115). Data include only indexes having at least one alter who returned a verified test result.

## Abbreviations

aRR: adjusted rate ratio
CBO: community-based organization
MSM: men who have sex with men
POL: popular opinion leader
STI: sexually transmitted infection

## References

[1] Borgatti SP, Mehra A, Brass DJ, Labianca G (2009). Network analysis in the social sciences. Science.

[2] Wang K, Brown K, Shen S, Tucker J (2011). Social network-based interventions to promote condom use: a systematic review. AIDS Behav.

[3] Harling G, Tsai AC (2019). Using social networks to understand and overcome implementation barriers in the global HIV response. J Acquir Immune Defic Syndr.

[4] Kelly JA, Murphy DA, Sikkema KJ, McAuliffe TL, Roffman RA, Solomon LJ, Winett RA, Kalichman SC (1997). Randomised, controlled, community-level HIV-prevention intervention for sexual-risk behaviour among homosexual men in US cities. Community HIV Prevention Research Collaborative. Lancet.

[5] Amirkhanian YA, Kelly JA, Kabakchieva E, Kirsanova AV, Vassileva S, Takacs J, DiFranceisco WJ, McAuliffe TL, Khoursine RA, Mocsonaki L (2005). A randomized social network HIV prevention trial with young men who have sex with men in Russia and Bulgaria. AIDS.

[6] Ko N, Hsieh C, Wang M, Lee C, Chen C, Chung A, Hsu S (2013). Effects of Internet popular opinion leaders (iPOL) among Internet-using men who have sex with men. J Med Internet Res.

[7] Young SD, Cumberland WG, Nianogo R, Menacho LA, Galea JT, Coates T (2015). The HOPE social media intervention for global HIV prevention in Peru: a cluster randomised controlled trial. Lancet HIV.

[8] Young SD, Holloway I, Jaganath D, Rice E, Westmoreland D, Coates T (2014). Project HOPE: online social network changes in an HIV prevention randomized controlled trial for African American and Latino men who have sex with men. Am J Public Health.

[9] Lightfoot MA, Campbell CK, Moss N, Treves-Kagan S, Agnew E, Kang Dufour MS, Scott H, Saʼid AM, Lippman SA (2018). Using a social network strategy to distribute HIV self-test kits to African American and Latino MSM. J Acquir Immune Defic Syndr.

[10] Lippman SA, Lane T, Rabede O, Gilmore H, Chen YH, Mlotshwa N, Maleke K, Marr A, McIntyre JA (2018). High acceptability and increased HIV-testing frequency after introduction of HIV self-testing and network distribution among South African MSM. J Acquir Immune Defic Syndr.

[11] Masters SH, Agot K, Obonyo B, Napierala Mavedzenge S, Maman S, Thirumurthy H (2016). Promoting partner testing and couples testing through secondary distribution of HIV self-tests: a randomized clinical trial. PLoS Med.

[12] Thirumurthy H, Masters SH, Mavedzenge SN, Maman S, Omanga E, Agot K (2016). Promoting male partner HIV testing and safer sexual decision making through secondary distribution of self-tests by HIV-negative female sex workers and women receiving antenatal and post-partum care in Kenya: a cohort study. Lancet HIV.

[13] Choko AT, Nanfuka M, Birungi J, Taasi G, Kisembo P, Helleringer S (2018). A pilot trial of the peer-based distribution of HIV self-test kits among fishermen in Bulisa, Uganda. PLoS One.

[14] Gichangi A, Wambua J, Mutwiwa S, Njogu R, Bazant E, Wamicwe J, Wafula R, Vrana CJ, Stevens DR, Mudany M, Korte JE (2018). Impact of HIV self-test distribution to male partners of ANC clients: results of a randomized controlled trial in Kenya. J Acquir Immune Defic Syndr.

[15] Campbell CK, Lippman SA, Moss N, Lightfoot M (2018). Strategies to increase HIV testing among MSM: a synthesis of the literature. AIDS Behav.

[16] Shelton RC, Lee M, Brotzman LE, Crookes DM, Jandorf L, Erwin D, Gage-Bouchard EA (2019). Use of social network analysis in the development, dissemination, implementation, and sustainability of health behavior interventions for adults: A systematic review. Soc Sci Med.

[17] Wu D, Tang W, Lu H, Zhang TP, Cao B, Ong JJ, Lee A, Liu C, Huang W, Fu R, Li K, Pan SW, Zhang Y, Fu H, Wei C, Tucker JD (2019). Leading by example: web-based sexual health influencers among men who have sex with men have higher HIV and syphilis testing rates in China. J Med Internet Res.

[18] Simoni JM, Franks JC, Lehavot K, Yard SS (2011). Peer interventions to promote health: conceptual considerations. Am J Orthopsychiatry.

[19] Kelly JA (2004). Popular opinion leaders and HIV prevention peer education: resolving discrepant findings, and implications for the development of effective community programmes. AIDS Care.

[20] Wu D, Zhou Y, Yang N, Huang S, He X, Tucker J, Li X, Smith KM, Ritchwood T, Jiang X, Liu X, Wang Y, Huang W, Ong J, Fu H, Bao H, Pan S, Dai W, Tang W (2020). Social media-based secondary distribution of HIV/syphilis self-testing among Chinese men who have sex with men. Clin Infect Dis.

[21] Li X, Fang X, Lin D, Mao R, Wang J, Cottrell L, Harris C, Stanton B (2004). HIV/STD risk behaviors and perceptions among rural-to-urban migrants in China. AIDS Educ Prev.

[22] Li X, Lu H, Ma X, Sun Y, He X, Li C, Raymond HF, McFarland W, Sun J, Pan SW, Shao Y, Vermund SH, Xiao Y, Ruan Y, Jia Y (2012). HIV/AIDS-related stigmatizing and discriminatory attitudes and recent HIV testing among men who have sex with men in Beijing. AIDS Behav.

[23] Feng Y, Wu Z, Detels R, Qin G, Liu L, Wang X, Wang J, Zhang L (2010). HIV/STD prevalence among men who have sex with men in Chengdu, China and associated risk factors for HIV infection. J Acquir Immune Defic Syndr.

[24] Okoboi S, Lazarus O, Castelnuovo B, Nanfuka M, Kambugu A, Mujugira A, King R (2020). Peer distribution of HIV self-test kits to men who have sex with men to identify undiagnosed HIV infection in Uganda: A pilot study. PLoS One.

[25] Tun W, Vu L, Dirisu O, Sekoni A, Shoyemi E, Njab J, Ogunsola S, Adebajo S (2018). Uptake of HIV self-testing and linkage to treatment among men who have sex with men (MSM) in Nigeria: A pilot programme using key opinion leaders to reach MSM. J Int AIDS Soc.

[26] Das A, George B, Ranebennur V, Parthasarathy MR, Shreenivas GS, Todankar P, Shrivastav A, Reddy AK, Akolo C, Cassell M, Mane S, Tripathi D, Baishya J (2019). Getting to the first 90: incentivized peer mobilizers promote HIV testing services to men who have sex with men using social media in Mumbai, India. Glob Health Sci Pract.

[27] Valente TW, Pumpuang P (2007). Identifying opinion leaders to promote behavior change. Health Educ Behav.

[28] Aral S, Muchnik L, Sundararajan A (2013). Engineering social contagions: Optimal network seeding in the presence of homophily. Netw Sci.

