# Supplementary material for: Sexual Health Influencer Distribution of HIV/Syphilis Self-Tests Among Men Who Have Sex With Men in China: Secondary Analysis to Inform Community-Based Interventions
Source: J Med Internet Res. 2021 Jun 1;23(6):e24303. doi: 10.2196/24303 (PMC8207256; doi:10.2196/24303)
Supplement: Multimedia Appendix 1 [file jmir_v23i6e24303_app1.docx]

**Multimedia Appendix 1. The six-item sexual health influencer scale; a higher score indicates greater influence**

| **Scale Item** | **Score** |
| --- | --- |
| How frequently do you discuss HIV/STI related topics with others? | 1= never, 2, 3, 4, 5 = very often |
| When you discuss HIV/STI related topics with others, how much information do you provide? | 1= almost none, 2, 3, 4, 5 = a lot |
| In the last 3 months, with how many people did you discuss HIV/STI related topics? | 1= none, 2, 3, 4, 5 = a lot |
| How likely are others in your social network to seek advice from you about HIV/STIs? | 1 = very unlikely, 2, 3, 4, 5 = very likely |
| When you discuss HIV/STIs with others in your network, what of the following is the most likely scenario? | 1 = you always give others information, 2, 3, 4, 5 = others always give you information |
| When you discuss HIV/STIs with others, how frequently do they seek advice from you? | 1= never, 2, 3, 4, 5 = very often |
