# Supplementary material for: Sexual Health Influencer Distribution of HIV/Syphilis Self-Tests Among Men Who Have Sex With Men in China: Secondary Analysis to Inform Community-Based Interventions
Source: J Med Internet Res. 2021 Jun 1;23(6):e24303. doi: 10.2196/24303 (PMC8207256; doi:10.2196/24303)
Supplement: Multimedia Appendix 3 [file jmir_v23i6e24303_app3.docx]

| **Total count (X)** | **Sexual health influencers**  **(I=77)** | | **Non-influencers (N=294)** | | **Influencer to non-influencer adjusted rate ratio,**  **aRR = R_I_/R_N_ (95% CI)^a^** |
| --- | --- | --- | --- | --- | --- |
|  | **Count for influencers**  **(i)** | **Rate per influencer**  **(R_I_ = i/I)** | **Count for non-influencers (n)** | **Rate per non-influencer**  **(R_N_=n/N)** |  |
| Number of applications (X=645) | 182 | 2.36 | 463 | 1.57 | 1.32 (1.10-1.58) |
| Total test kits requested (X=1148) | 344 | 4.47 | 804 | 2.73 | 1.40 (1.22-1.60) |
| Alters who returned a self-test result (X=278) | 128 | 1.66 | 150 | 0.51 | 2.07 (1.59-2.69) |
| ^a^ Controlled for index income, disclosure status, volunteer status, and prior HIV testing | | | | | |
