# Supplementary material for: Sexual Health Influencer Distribution of HIV/Syphilis Self-Tests Among Men Who Have Sex With Men in China: Secondary Analysis to Inform Community-Based Interventions
Source: J Med Internet Res. 2021 Jun 1;23(6):e24303. doi: 10.2196/24303 (PMC8207256; doi:10.2196/24303)
Supplement: Multimedia Appendix 4 [file jmir_v23i6e24303_app4.docx]

|  | **Sexual health influencers (I=36)** | | **Non-influencers (N=80)** | |  | **Influencer to non-influencer adjusted**  **rate ratio,**  **aRR = R_I_/R_N_ (95% CI)^a^** |  |  |
| --- | --- | --- | --- | --- | --- | --- | --- | --- |
| **Total count (X)** | **Count for influencers (i)** | **Rate per influencer (R_I_=i/I)** | **Count for non-influencers (n)** | **Rate per non-influencer (R_N_=n/N)** | **Influencer to non-influencer rate ratio,**  **RR = R_I_/R_N_ (95% CI)** |  |  |  |
| Alters tested first time for HIV (X=106) | 44 | 1.22 | 62 | 0.78 | 1.58  (1.07-2.32) | 1.28  (0.85-1.92) |  |  |
| Alters tested simultaneously with index (X=138) | 51 | 1.42 | 87 | 1.09 | 1.30  (0.92-1.84) | 1.16  (0.81-1.66) |  |  |
| Alters with HIV reactive result (X=15) | 8 | 0.22 | 7 | 0.09 | 2.54  (0.92-7.00) | 2.37  (0.83-6.76) |  |  |
| Alters with syphilis reactive result (X=8) | 4 | 0.11 | 4 | 0.05 | 2.22  (0.56-8.89) | 1.91  (0.45-8.12) |  |  |
|  | | | | | | | |  |
| ^a^ Controlled for index CBO volunteer status | | | | | | | | |
