# Supplementary material for: Sexual Health Influencer Distribution of HIV/Syphilis Self-Tests Among Men Who Have Sex With Men in China: Secondary Analysis to Inform Community-Based Interventions
Source: J Med Internet Res. 2021 Jun 1;23(6):e24303. doi: 10.2196/24303 (PMC8207256; doi:10.2196/24303)
Supplement: Multimedia Appendix 5 [file jmir_v23i6e24303_app5.docx]

| **Total count (X)** | **Sexual health influencers (I=77)** | | **Non-influencers (I=294)** | | **Influencer to non-influencer adjusted rate ratio,**  **aRR = R_I_/R_N_ (95% CI)^b^** | |
| --- | --- | --- | --- | --- | --- | --- |
|  | **Count for influencers**  **(i)** | **Rate per influencer**  **(R_I_ = i/I)** | **Count for non-influencers (n)** | **Rate per non-influencer**  **(R_N_=n/N)** |  |  |
| Number of applications (X=616) | 153 | 2.01 | 463 | 1.57 | 1.22 (1.01-1.48) | |
| Total self-tests requested (X=1102) | 298 | 3.92 | 804 | 2.73 | 1.32 (1.15-1.52) | |
| Alters who returned a self-test result^a^ (X=241) | 91 | 1.20 | 150 | 0.51 | 1.80 (1.35-2.38) | |
| ^a^ Defined as unique alters who returned a verified test result | | | | |  |  |
| ^b^ Controlled for index income, disclosure status, volunteer status, and prior HIV testing | | | | | |  |
