# Supplementary material for: Sexual Health Influencer Distribution of HIV/Syphilis Self-Tests Among Men Who Have Sex With Men in China: Secondary Analysis to Inform Community-Based Interventions
Source: J Med Internet Res. 2021 Jun 1;23(6):e24303. doi: 10.2196/24303 (PMC8207256; doi:10.2196/24303)
Supplement: Multimedia Appendix 6 [file jmir_v23i6e24303_app6.docx]

| **Total count (X)** | **Sexual health influencers (n=35)** | | **Non-influencers (n=80)** | | **Influencer to non-influencer adjusted rate ratio (95% CI)^a^** |
| --- | --- | --- | --- | --- | --- |
|  | **Count for influencers (i)** | **Rate per influencer (R_I_=i/I)** | **Count for non-influencers (n)** | **Rate per non-influencer (R_N_=n/N)** |  |
| Alters tested first time for HIV (X=91) | 29 | 0.83 | 62 | 0.78 | 1.01 (0.64-1.59) |
| Alters tested simultaneously with index (X=132) | 45 | 1.29 | 87 | 1.09 | 1.10 (0.76-1.59) |
| Alters with HIV reactive result (X=15) | 8 | 0.2 | 7 | 0.09 | 2.25 (0.77-6.58) |
| Alters with syphilis reactive result (X=8) | 4 | 0.11 | 4 | 0.05 | 1.95 (0.46-8.26) |

^a^Controlled for index income, disclosure status, volunteer status, and prior HIV testing.
