# Supplementary material for: Sexual Health Influencer Distribution of HIV/Syphilis Self-Tests Among Men Who Have Sex With Men in China: Secondary Analysis to Inform Community-Based Interventions
Source: J Med Internet Res. 2021 Jun 1;23(6):e24303. doi: 10.2196/24303 (PMC8207256; doi:10.2196/24303)
Supplement: Supplementary file 6 [file jmir_v23i6e24303_app2.docx]

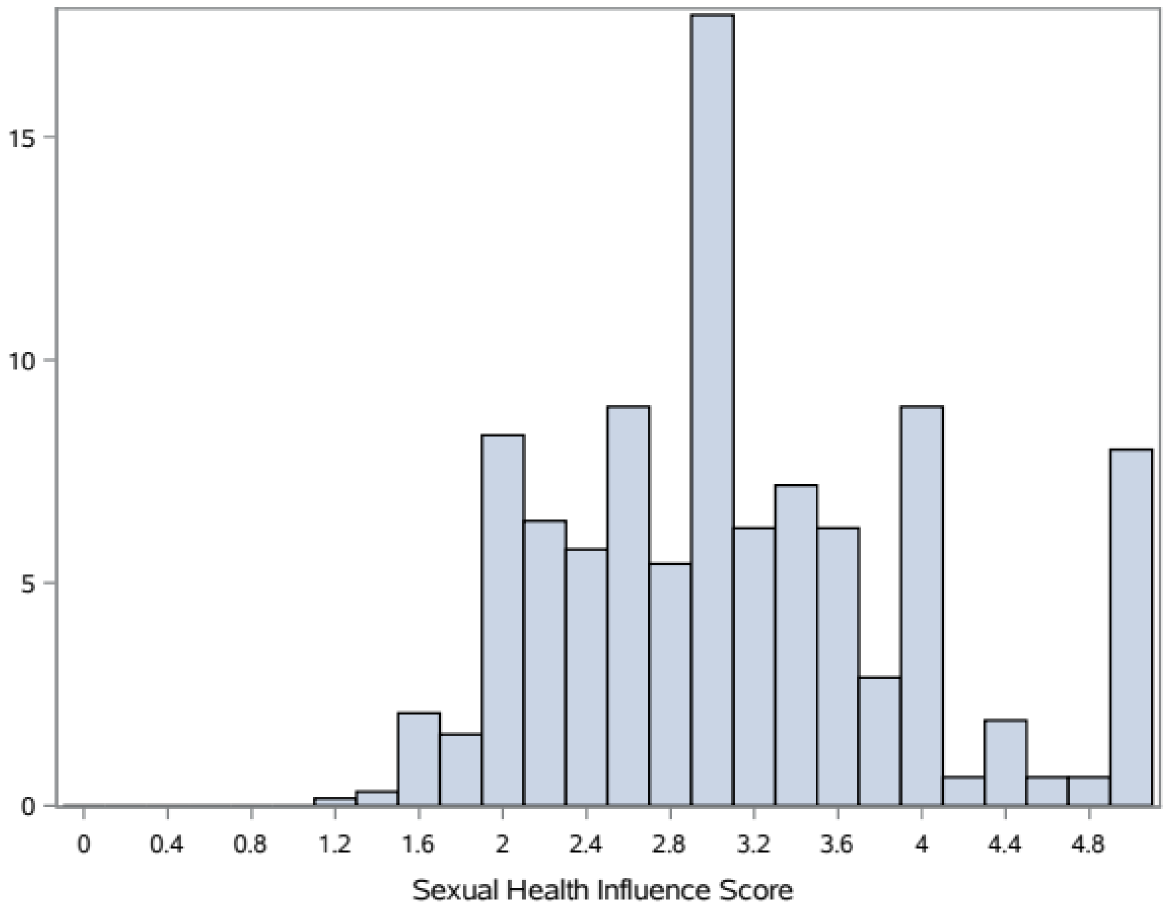
15
10
5
0
0
0.4
0.8
1.2
1.6
2
2.4
2.8
3.2
3.6
4
4.4
4.8
Sexual Health Influence Score
